# Discussion to: Outcomes and residual gap analysis after the modified cryomaze procedure performed via right minithoracotomy versus sternotomy

**DOI:** 10.1016/j.xjon.2023.07.018

**Published:** 2023-07-28

See Article page 176.

Presenter: Dr Takashi Kakuta

**Unidentified Speaker 1**. Dr Richard Whitlock from McMaster University will discuss the paper.

**Dr Richard Whitlock***(Hamilton, Ontario, Canada)*. Great. Congratulations to Dr Kakuta. Dr Kakuta actually contacted me while I was in Japan. I had the pleasure of visiting your country and have to say it's amazing. I would promote everybody here to go and visit. The surgeons were very kind to me, and I also ran the Tokyo marathon, which was fantastic. So could you describe to us—there clearly was a shift in terms of your technique to try to mitigate this risk, which I think was fantastic to identify. Whenever we shift practice away from a gold standard open sternotomy full lesion set, we have to understand what we're doing. When you changed to your vertical incision in the right atrium, what does the proportion of gaps look like after you made that change?

**Figure:**
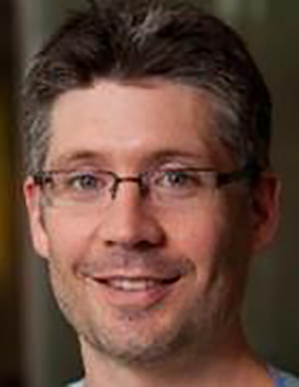

**Dr Takashi Kakuta***(Osaka, Japan)*. Thank you for your great question. In the right minithoracotomy group, 70 patients had a transverse incision and 70 patients had a vertical incision and only 2 patients had an additional ablation in the vertical line group, and there were no gaps at the tricuspid line. So, the case number was so small, so it's hard to be definite, but we believe that this modification contributes to the improvement of the outcome to the right minithoracotomy. Thank you.

**Figure:**
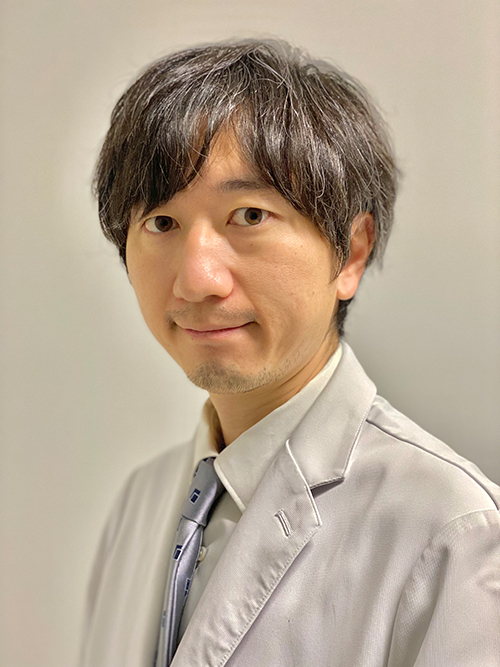

**Dr Whitlock**. Second question is, so your right mini-thoracotomy group actually had more left atrial appendage occlusion than your sternotomy group, and yet it's easier to do and probably more effective than the sternotomy approach. Was it different surgeons doing it? Why was that?

**Dr Kakuta**. Thank you. Previously, in our institution, we saw the preservation of left atrial appendage contributes to maintain cardiac function by atrial kick or hormone. So, in some cases, some patients didn't have the atrial appendage occlusion, but recently the evidence was established. So, we perform the left atrial appendage occlusion in all cases.

**Dr Whitlock**. Yeah, I heard that amongst some of the Tokyo surgeons too, but they have now shifted to everybody gets their appendage managed, which makes me happy. The failure in the mitral line in the sternotomy cases—and yet, it was not failing in your right minithoracotomy cases. What's your hypothesis around that? And what have you done to rectify that?

**Dr Kakuta**. Thank you. To answer that question, I have to mention the coronary sinus ablation. Honestly, we didn't perform the coronary sinus ablation from the [inaudible] side, but we changed strategy as in 2018, we started to perform the coronary sinus ablation from that side in all cases. So, it might affect the distribution of the residual gap. So, and also, it might affect the improvement of the outcomes in the sternotomy group.

**Dr Whitlock**. Last is more of a comment. So, when I first looked at the data in the baseline characteristics, I thought, oh man, this is a big imbalance here in terms of risk factors for performance of this procedures. And it looked like the right mini-thoracotomy procedure should perform better based on the characteristics. But in fact, it didn't. So, I think your findings certainly are real and also, I think the most important thing, again, is the gap analysis in it, so fantastic work and thank you so much.

**Dr Kakuta**. Thank you.

**Unidentified Speaker 1**. Ralph.

**Dr Ralph Damiano***(St Louis, Mo)*. Ralph Damiano, St Louis. First of all, congratulations on a great presentation and also congratulations on looking at the residual gaps. We often don't get to do that. And I think that does add something. But I would really take a little issue with your conclusion. I don't think you can—it's your right minithoracotomy approach was not as good as sternotomy approach. We've been doing right minithoracotomy both, and we do it as a procedure of choice for all mitral and tricuspid surgery and for certainly all lone mazes since 2002. And we just published 10-year outcomes, and actually, right minithoracotomy was a risk factor for success, actually, for restoration. The restoration was a little higher. We did have problems in our study. But I don't really think there's any difference between the approaches. There's a difference if you can't see. So that would be more, and you've altered that. But it has really nothing to do—I mean, visualization can be challenging in a minithoracotomy, and you showed your learning curve. But I don't think—and certainly, in our center, it's been the opposite. I had just a couple of questions. First of all, I think it's among the first studies I've ever seen that showed that a minimally invasive approach was faster than a sternotomy approach. I'm interested in—your procedural times were less than your mini-thoracotomy group. I'm not sure I've ever seen any paper in our field that has shown that. So, I'm interested in that if you could comment on that.

**Figure:**
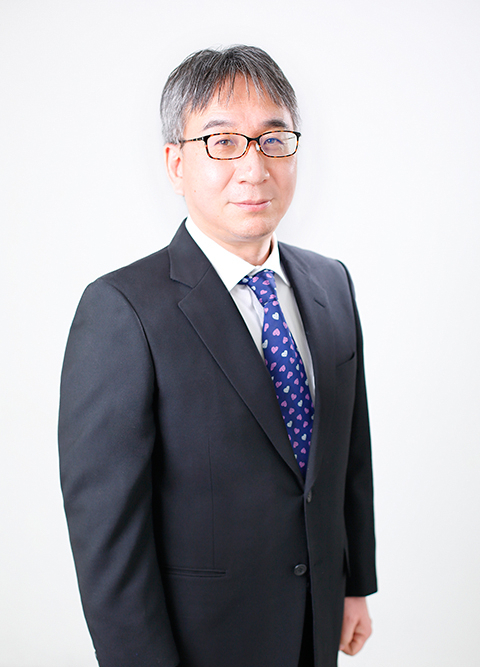

And number 2 is, I do think exposure is critical. And in a minithoracotomy, though, with a scope, you can really see, and I noticed you used the robot, so you should have had really good visualization. But I think it's more—I would agree with you. If you're using cryo only, in my opinion, and when we've done it, I mean, you have to be a little more careful with those long probes that you don't get gaps. And I think you could even see in your thing, because of some of the trabeculation and the right atrium, if you don't really flatten the tissue out and get excellent contact the whole length of that probe, you're going to get gaps. And maybe you were just a little bit better in the sternotomy and in being able to flatten the tissue out and avoid—because even the smallest gap, less than a millimeter between the cryoprobe and the tissue is going to result in an incomplete lesion. I wonder if you could comment on those 2 things. One, how you were so fast with the minimally invasive, and then what other techniques to avoid gaps when you're working through a minithoracotomy.

**Dr Kakuta**. Thank you. At first, 66% of the right mini-thoracotomy was concomitantly performed with the robotic mitral valve repair. And in the institution, the operation time of the robotic mitral valve repair is significantly shorter than the sternotomy approach. And also, we checked why the operation time was shorter in the mini-thoracotomy. And we hemostasis time and hemostasis time was really shorter in the right minithoracotomy. In total, the right minithoracotomy has a shorter operation time. And yeah, as you mentioned, the—

**Unidentified Speaker 2**. I just wanted to follow up on that. Could it be a surgeon difference? Did you have the same surgeons doing both the sternotomy and the mini-thoracotomy?

**Dr Kakuta**. Eighty-five percent of this procedure is performed by only 1 surgeon. Robotic surgeon is main reason of the good [inaudible] right minithoracotomy, I guess. Depending on the surgeon, it's very important to [inaudible]. And another good thing about this robotic procedure, if we are not sufficiently contact the tissue, the cryo operation, the surgeon assists the bedside surgeon to contact well to the tissue, exposing the tissue, and does not overlap as well. And also assist of the contact by the robot, we've done it. So yeah, great thing of the robotic procedure.

**Unidentified Speaker 1**. One more quick question, Hal Roberts.

**Dr Hal Roberts***(St Louis, Mo)*. Again, just Hal Roberts from St Louis. I just wanted to ask in as far as your technique. If I read that, saw that correctly, you had a minute-and-a-half ablation time 90 seconds. Is that correct? How long was your ablation time?

**Dr Kakuta**. Ablation time is 90 seconds.

**Dr Roberts**. Which power source were you using?

**Dr Kakuta**. I'm sorry?

**Dr Roberts**. Which power source, argon, or nitrous?

**Dr Kakuta**. Nitrous

**Dr Roberts**. Nitrous? Well, I would proffer that perhaps you weren't ablating long enough. 90 seconds, I think a minimum of 2 minutes, I think, is what most guys do and perhaps longer. With argon, for the lesions, I can't see along the entire track. I ablate 3 minutes even though I know it's overkill. And I would just offer that as a potential criticism as to why your data are not that great actually with either approach.

**Unidentified Speaker 1**. Thank you very much.

